# Impact of Player Injuries on Teams' Mental States, and Subsequent Performances, at the Rugby World Cup 2015

**DOI:** 10.3389/fpsyg.2016.00807

**Published:** 2016-06-03

**Authors:** Olivia A. Hurley

**Affiliations:** Department of Technology and Technology, Faculty of Film, Art and Creative Technologies, Dun Laoghaire Institute of Art, Design and TechnologyDublin, Ireland

**Keywords:** rugby, challenges, injury, social contagion, team ethos, equality, technology, virtual reality

Injuries are considered an inevitable by-product of participation in elite sport (Hurley et al., 2007), and over the past 30 years, psychologists have proposed a number of models to explain athletes' psychological reactions to their injuries. These models have included the grief model (Kübler-Ross, 1969; Rotella, 1988) and the cognitive appraisal models (Brewer, 1998; Wiese-Bjornstal et al., 1998). When examining the value of these models, researchers have identified a number of factors mediating athletes' responses to their injuries. Factors such as social support (Clement and Shannon, 2011) and perceived consequences of injuries for the athletes (Hurley et al., 2007) have been popular lines of investigation. A less popular avenue of research has been the impact of tournament-ending injuries on the mental states of remaining squad members called upon to perform without their injured colleagues. Given the lack of research in this area, it could be fruitful to discuss the potential impact of player attrition rates on their teams' mental states, and subsequent performances, using the Rugby World Cup 2015 (RWC 2015) as a case study. This case study is considered appropriate because many countries suffered serious injuries to some of their best players just before, or during, this RWC (Lewis, 2015). For example, the Irish team lost one of its most experienced players, Paul O'Connell, due to injury early in the tournament, as did South Africa, who was forced to play without captain, Jean De Villiers, from game three of the tournament. Similarly, the New Zealand “All Blacks” lost their experienced player, Tony Woodcock, due to injury before their quarter final match. Despite the loss of Woodcock, the New Zealand team was successful in defending its RWC title from 2011. Indeed in 2011, New Zealand played without four of its out-half players. Is it possible the New Zealand team did something different, compared to the other nations, to prepare successfully for both the RWC 2011 and 2015 tournaments? This paper will explore possible reasons why some of the other nations failed to perform to their potential at the RWC 2015.

This paper is organized as follows: First, the mental challenges team players may have faced following the loss of influential players through injury will be discussed. The potential for emotional contagion to have permeated throughout such teams, and its possible impact on those teams' subsequent performances will dominate this discussion (Moll et al., 2010). Second, some possible areas for future research will be suggested, focusing on the role support staff (including coaches, medical staff, and sport psychologists) working with such teams might play in helping their teams to thrive in such circumstances in the future.

## Possible mental challenges for team players at the RWC 2015, following the loss of influential team members through injury

As mentioned earlier, many teams competing at RWC 2015 suffered the loss of influential players due to injury during the warm-up, pool, and knock-out stages of the tournament (Lewis, 2015). In rugby union, which is a collision sport, the potential for injury is high when compared to non-contact sports (Almedia et al., 2014). Tournament-ending injuries to such elite team players have the potential to cause disturbances not only in the injured players' emotional and behavioral states, but also in the remaining team members' mental states and on-pitch performances. Seeing a teammate suffer a tournament-ending injury could result in other team members experiencing an increased fear of injury themselves, and decreased levels of confidence in the team's overall ability to continue to perform at a high level in the competition. Indeed, the Head Coach of South Africa, Heyneke Meyers, commented that De Villiers was the “glue” of the South African team and that his influence would be missed (The Guardian, 2015). It is interesting then that empirical research measuring the impact of such player losses has not been the focus of researchers' attention to date, despite common sense dictating that such losses could contribute to changes in teams' collective thought processes at competitions such as the RWC (Cary, 2015). For example, doubts surrounding the ability of another player to successfully perform in the role of an injured colleague within a team could have occurred at the RWC 2015. This potential for negative social contagion to develop within teams who suffer high numbers of player losses due to injury is worthy of attention.

Social contagion refers to the spread of mood states, attitudes or behaviors from an initial person, called “the initiator,” to another person, called “the recipient,” without the recipient being necessarily aware of this effect of the initiator (Levy and Nail, 1993, p. 266). In general, studies of social contagion have focused on two main areas (i) emotional contagion—the spread of mood states throughout a population, and (ii) behavioral contagion—the spread of behaviors throughout a population. Some empirical studies of social contagion have been conducted in real-world, high-performance settings. For example, Wagstaff and Weston (2014) examined the impact of social contagion on teams making expeditions to the Antarctica. Interestingly, some of the negative contagion-performance effects reported by Wagstaff and Weston (2014) have also been reported in environments where direct contact between individuals has not taken place, such as through social media outlets (i.e., Facebook; see Kramer et al., 2014). These findings highlight that contagion effects in performance settings should not be underestimated.

Emotional contagion implies that emotions expressed by one individual in a group or team, may spread to other members of that collective group. Some factors thought to be important in determining the rate and extent of emotional contagion, according to Bartel and Saavedra (2000), include: (i) the degree of membership stability within the group, i.e., the turnover rate of members within the group, and the number of years members are part of that group (Squire, 1988), (ii) the task interdependence within the group, i.e., how much group members rely on one another to complete their tasks effectively (Georgopoulos, 1986), and (iii) the social interdependence within the group, i.e., the impact of other group members' actions on other individuals' abilities to accomplish their goals [such impacts may be positive (cooperative) or negative (competitive); Johnson and Johnson, 2008]. Some coaches and support staff of RWC 2015 teams would, perhaps, have benefited from having a greater understanding of the impact of such factors on their teams' performances.

Often, during the RWC 2015, spectators, commentators, and coaches discussed the potential impact of injuries on teams' performances (Hewett, 2015; The Guardian, 2015). On occasion, they questioned the ability of substitute players to effectively replace their injured teammates (Cary, 2015). The lack of research on the potential impact of team members' reduced confidence in a replacement player's ability exposes an area for investigation. Perhaps participants in such studies may be viewed as reluctant to voice their real concerns about fellow team members being capable of replacing their unavailable injured colleagues. This view may account for researchers avoiding this specific avenue of investigation. The good news is that while individuals may be affected negatively by those around them, they may also be affected in a positive way (Fransen et al., 2015). This point prompts the question of what can be done to enable team members maintain high levels of confidence in their own, and other teammates', ability to perform successfully in their roles, when placed in high pressure situations such as those presented at the RWC 2015 (something the New Zealand team appear to have successfully achieved, as is evident from their recent successes in the tournament).

## The specific role support staff may play in helping teams, including replacement players, to thrive in high pressure competitions, such as the RWC

Team coaches, medical personnel, and sport psychologists may play an important role in helping team members to remain confident and thrive in situations where the loss of an influential player occurs, such as at the RWC. Rather than seeing the situation as a threat to success, players could be encouraged to see the situation as an accepted and positive challenge they are capable of overcoming (Hodge and Smith, 2014). One suggestion for support staff could be to spend more time, in advance of such tournaments, creating a team ethos centered on equality among all players and support staff. A number of recent studies have been devoted to the “teamwork” dimension of team cohesion (McEwan and Beauchamp, 2015; also see Moran, 2012, for a review of this topic). Perhaps, placing a greater emphasis on “team spirit” and player acceptance is warranted now in team environments, in order to positively impact on the character and attitudes of team members. This appears to be something the New Zealand players have been successful in achieving in recent years, based on their results at the RWC 2011 and 2015. Coaches and support staff who actively promote and encourage an investment in personal relationships among all members of their team environment are more likely to develop a greater team awareness of the importance of interpersonal dynamics. Such an approach to team management could increase the sense of equality and acceptance within the team (Garn, 2016). It may also help to create an attitude of personal responsibility for performances and a greater acceptance of individual leadership duties by all players within a team (Kerr, 2013; Smith, 2015).

One novel way to enhance players' coping skills in times of unrest and stress due to player injury losses might involve the use of virtual reality technology, such as the Oculus Rift (Oculus, 2016). Virtual match-day scenarios could be designed to simulate match-day events, where key players are made “unavailable due to injury,” and other players are required to “replace” them. Programs could be designed to prepare all available players, including replacement players, for the demands and atmosphere of such match-day possibilities. Players could be taught to engage in relaxation and concentration strategies (i.e., self-talk management, appropriate goal setting, breathing exercises, and progressive muscular relaxation techniques; Cotterill, 2015) while watching such match-day simulations, in order to train them to remain calm, focused, and positive in their mind-set when called upon to perform at important tournaments. These match simulations could be used by all players well in advance of important tournaments to help them mentally prepare for these possible real-life events (Pramuk, 2016). Researchers could also empirically investigate if such virtual preparation compared to, or along with, for example, mental imagery rehearsal of such scenarios, could establish greater player confidence and acceptance for all players' contributions within a squad of players. Similar virtual reality simulations have been used successfully with stroke patients (Hurley, 2016) and to prepare injured players for a return to play (Independent Pictures, 2013). Therefore, the use of such technology would not be unfamiliar to many elite players.

## Final thoughts

It is possible teams who, by their own standards, did not produce peak performances at the RWC 2015 suffered the negative effects of emotional, and behavioral, contagion due to player injuries. As discussed above, these outcomes may have occurred as a result of decreased levels of confidence in replacement team members' potential performances, although such assertions are in need of more support via empirical investigations. How such situations may be prevented from hampering teams' performances in future competitions should also be the focus of empirical research by psychologists working with these teams. Such research might help to provide a greater understanding of the unexpected decreases in some teams' performances at the RWC 2015 (Boss and Kleinert, 2015). Perhaps engaging in mental skills training using a virtual reality environment could inoculate players against the negative emotional and performance effects of social contagion. One should also consider that tournaments such as the RWC occur in 4-year cycles, therefore for such an intervention to be effective, the club environments of players should also incorporate a similar approach to players' preparations.

## Author contributions

The author confirms being the sole contributor of this work and approved it for publication.

### Conflict of interest statement

The author declares that the research was conducted in the absence of any commercial or financial relationships that could be construed as a potential conflict of interest.
