# Corrigendum: Impact of Player Injuries on Teams' Mental States, and Subsequent Performances, at the Rugby World Cup 2015

**DOI:** 10.3389/fpsyg.2016.01127

**Published:** 2016-07-27

**Authors:** Olivia A. Hurley

**Affiliations:** Department of Technology and Psychology, Faculty of Film, Art and Creative Technologies, Dun Laoghaire Institute of Art, Design and TechnologyDublin, Ireland

**Keywords:** rugby, challenges, injury, social contagion, team ethos, equality, technology, virtual reality

Reason for Corrigendum:

There was a mistake in the author's affiliation as published in the Opinion article. The correct affiliation is Department of Technology and Psychology and not Department of Technology and Technology as reported.

The author apologizes for the mistake. This error does not change the content or opinions put forward in the opinion article as published.

## Author contributions

The author confirms being the sole contributor of this work and approved it for publication.

### Conflict of interest statement

The author declares that the research was conducted in the absence of any commercial or financial relationships that could be construed as a potential conflict of interest.

